# The Solute Carrier (SLC) Transporter Superfamily as Therapeutic Targets for the Treatment of Head and Neck Squamous Cell Carcinoma

**DOI:** 10.3390/cancers16183226

**Published:** 2024-09-22

**Authors:** Sang Yeon Cho, Nam Sook Kang

**Affiliations:** 1 Graduate School of New Drug Discovery and Development, Chungnam National University, Daejeon 34134, Republic of Korea; nprc26@cnu.ac.kr; 2 CHOMEDICINE Inc., TIPS Town, Chungnam National University, Daejeon 34135, Republic of Korea

**Keywords:** SLC superfamily, head and neck squamous cell carcinoma, precision oncology

## Abstract

**Simple Summary:**

Head and neck squamous cell carcinoma (HNSC) is the most common type of cancer in the head and neck region, affecting areas like the mouth and throat. Understanding how these cancers grow and spread is crucial for developing better treatments. This study focuses on a group of proteins called solute carrier (SLC) transporters, which help cancer cells survive and grow by moving nutrients and other molecules in and out of cells. We investigated the levels of these transporters in cancerous tissues compared to normal tissues and found that certain SLC transporters are much more active in tumors. These transporters are linked to worse outcomes for patients. By targeting these specific transporters with new drugs, we hope to create more effective treatments that can block cancer growth and improve survival rates for patients with HNSC.

**Abstract:**

**Background**: Head and neck squamous cell carcinoma (HNSC) is the most prevalent cancer in the head and neck region, originating from the mucosal epithelium of the oral cavity, pharynx, and larynx. The solute carrier (SLC) transporter superfamily, consisting of over 400 proteins across 65 families, plays a crucial role in cellular functions and presents promising targets in precision oncology. This study aims to analyze the expression of SLC transporters in HNSC and their potential as biomarkers and therapeutic targets. **Methods**: We leveraged mRNA and protein expression data from The Cancer Genome Atlas (TCGA) and The Human Protein Atlas (HPA) to examine SLC transporter expression in HNSC. Gene Set Enrichment Analysis (GSEA) was conducted to assess the involvement of SLC transporters in various oncogenic pathways. **Results**: Significant upregulation of SLC transporters was observed in tumor tissues compared to normal tissues, with notable increases in SLC16A3, SLC53A1, SLC25A32, and SLC2A3. This upregulation correlated with poorer overall survival (OS) and disease-specific survival (DSS). GSEA revealed that these transporters are significantly involved in critical oncogenic pathways, including epithelial-mesenchymal transition (EMT), angiogenesis, and hypoxia, which are vital for cancer progression and metastasis. **Conclusions**: The study identifies SLC transporters as potential biomarkers and therapeutic targets in HNSC. Targeting these transporters with small molecule inhibitors could disrupt essential supply routes for cancer cells, enhancing treatment efficacy and improving patient outcomes. This study paves the way for developing SLC-based target therapies in precision oncology, with the goal of improving survival rates for patients with HNSC.

## 1. Introduction

Head and neck squamous cell carcinoma (HNSC) is a highly aggressive malignancy, representing a significant challenge in oncology due to its complex molecular landscape [1]. Despite some association with risk factors like tobacco, alcohol, and human papillomavirus (HPV), the majority of HNSC cases are driven by intricate genetic and epigenetic alterations that disrupt normal cellular processes [2]. Recent genomic studies have uncovered a range of mutations, copy number variations, and epigenetic modifications that contribute to the initiation and progression of HNSC [3]. The genetic and epigenetic alterations of SLC transporters not only promote uncontrolled cell growth and invasion but also confer resistance to conventional therapies, highlighting the need for targeted therapeutic strategies [4,5,6].

Precision medicine, particularly precision oncology, aims to develop treatments tailored to individual genetic profiles and tumor characteristics, addressing novel therapeutic targets to improve patient outcomes [7]. Advances in genomic and proteomic technologies have enabled the identification of molecular alterations that drive tumor development, leading to the creation of targeted therapies that offer enhanced efficacy with reduced side effects [8,9]. The solute carrier (SLC) transporter superfamily, consisting of over 400 members organized into 65 families based on sequence homology and transport function, has emerged as a critical group of proteins involved in the transport of a wide range of substrates, including ions, metabolites, and drugs, across cellular membranes [10]. Given that many SLC transporters are expressed on the cell surface, they present promising targets for small molecule therapies [11]. These transporters are integral to cellular homeostasis, metabolic pathways, and signal transduction, making them attractive candidates for targeted cancer therapy [10,11]. Dysregulation of SLC transporters has been linked to various cancer-related processes such as proliferation, apoptosis, and metastasis. However, the comprehensive expression and functional implications of the SLC transporter superfamily in HNSC remain underexplored. While specific SLC transporters have been associated with poor prognosis in other cancers, their roles in HNSC have not been extensively studied [12]. Unraveling the complex roles of these transporters is essential for identifying new SLC targets and advancing the development of next-generation SLC-based therapeutics in precision oncology [13].

This study aims to provide a comprehensive analysis of the SLC transporter superfamily as potential therapeutic targets for the treatment of HNSC. By utilizing extensive mRNA and protein expression data from The Cancer Genome Atlas (TCGA-HNSC) and The Human Protein Atlas (HPA), along with functional enrichment analyses such as Gene Set Enrichment Analysis (GSEA), we systematically assess the differential expression, genetic alterations, and prognostic significance of SLC transporters in HNSC. Our findings reveal the crucial roles of specific SLC transporters in the pathophysiology of HNSC, highlighting their potential as biomarkers for diagnosis and prognosis, as well as viable targets for therapeutic intervention. This study emphasizes the significance of SLC transporters in cancer biology and their promise as candidates for developing novel therapeutic strategies in the management of HNSC.

## 2. Materials and Methods

### 2.1. The mRNA and Protein Expression Data

HNSC gene expression RNAseq and clinical information data, uniformly processed by the TOIL process to be free of computational batch effects, were downloaded from UCSC Xena (https://xenabrowser.net/datapages/?cohort=GDC TCGA Head and Neck Cancer (HNSC), accessed on 5 January 2024), including The Cancer Genome Atlas (TCGA) and Genotype-Tissue Expression (GTEx) data available on the platform [14]. The raw data were analyzed using R (v.4.3.3, http://www.r-project.org, accessed on 4 May 2024). cBioportal (http://www.cbioportal.org/, accessed on 5 May 2024) was also used to analyze the mRNA expression and to identify gene alterations in TCGA-HNSC [15]. All the protein expression and immunohistochemical staining data were obtained from The Human Protein Atlas (HPA) database (http://www.proteinatlas.org, accessed on 12 January 2024). Matched immunohistochemistry images and information were then used to compare and analyze the immunohistochemistry results for SLC genes using the R package “HPAanalyze” [16].

### 2.2. Kaplan-Meier Survival Analysis

To analyze the independent prognostic value of SLC superfamily members in HNSC, the Kaplan-Meier plot was used to display overall survival (OS) and disease-specific survival (DSS) based on RNA-seq expression data extracted from TCGA-HNSC by UCSC Xena. The optimal cutoff values for HNSC mRNA expression were determined using Cutoff Finder with the log-rank test [17]. A log-rank *p*-value <0.05 was considered statistically significant.

### 2.3. Structural Analysis of SLC Proteins

To analyze the structure of SLC proteins, we utilized the Alphafold database and AlphaMissense tools. The Alphafold database (https://alphafold.ebi.ac.uk/, accessed on 8 June 2024) provided high-confidence protein structure predictions based on deep learning algorithms, allowing us to obtain detailed 3D models of SLC transporters [18]. Additionally, we employed AlphaMissense to evaluate the potential impact of missense mutations on the protein structure and function. This tool integrates structural information with variant effect predictions, allowing for a comprehensive assessment of how specific amino acid changes might influence the activity and stability of SLC transporters [19].

### 2.4. Functional Enrichment Analysis

Gene Set Enrichment Analysis (GSEA) was employed to investigate the enrichment of mRNAs predicted to correlate with biological pathways within the hallmark and the Kyoto Encyclopedia of Genes and Genomes (KEGG) database, between groups characterized by high and low expression levels of the target SLC gene. This approach allowed for the identification of significant pathways that are differentially regulated between these two groups, providing insights into the underlying biological mechanisms. Statistical significance was assessed, with a threshold *p*-value of less than 0.05 deemed to indicate significant enrichment.

### 2.5. Statistical Analysis

Statistical analyses were conducted using R software, version 4.3.3 (available at http://www.r-project.org, accessed on 12 January 2024). Continuous variables were analyzed using the t-test or the Kolmogorov–Smirnov test if the expected frequency in any category was less than five. For categorical variables, the χ2 test or Fisher’s exact test was applied under the same conditions. When comparing characteristics across three or more groups, ANOVA was utilized. To address the issue of multiple comparisons, Bonferroni and Benjamini-Hochberg corrections were applied to *p*-values derived from the *t*-tests comparing 446 SLC genes between the normal and cancer groups. Statistical significance was established at a *p*-value of less than 0.05.

## 3. Results

### 3.1. Differential mRNA Expression of SLC Superfamily

**Figure 1:**
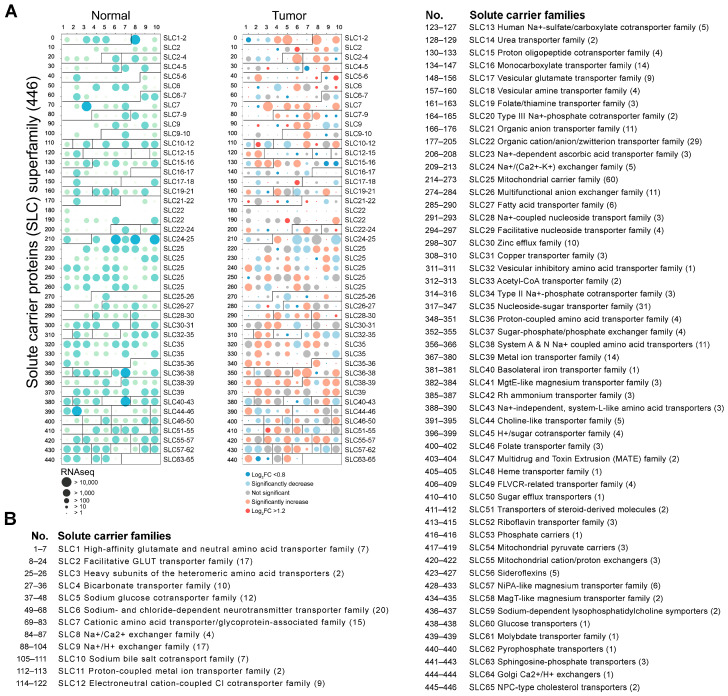
Differential expression of SLC superfamily genes in normal and tumor tissues. (**A**) The dot plot depicting the RNA-seq expression levels of 446 SLC superfamily in normal (**left panel**, green circles) and tumor tissues (**right panel**, red circles). The size of the circles corresponds to the RNA-seq expression levels, with larger circles indicating a higher expression. Significant changes in expression between normal and tumor tissues are indicated by the color intensity and size of the circles. (**B**) The list enumerates the 65 SLC families, providing the name for the specific SLC families analyzed and their corresponding SLC numbers.

To investigate the differential expression of 65 SLC families in normal and tumor tissues, we performed a comprehensive RNA-seq analysis covering 446 SLC genes in this study. All 446 SLC genes are summarized in Supplementary Table S1, categorized based on their sequence homology and transport function (http://slc.bioparadigms.org/, accessed on 8 September 2023). The results are detailed in Figure 1A, which illustrates the expression levels of these genes in both normal and tumor tissues. Notably, there are dramatic changes in the expression patterns of many SLC genes when comparing normal tissues to tumor tissues. Remarkably, 101 SLC genes exhibited a significant increase in expression, while 78 SLC genes showed a significant decrease. The top three SLC genes with the highest expression in normal head and neck tissue, in order, were SLC42A3, SLC25A6, and SLC25A3. Most notably, in HNSC, the top three SLC genes with the highest expression, in order, are SLC2A1, SLC7A5, and SLC38A2. Figure 1B enumerates the 65 SLC families, detailing the names of the specific SLC families analyzed along with their corresponding SLC numbers.

### 3.2. Prognostic Value of SLC Gene Expression on Patient Survival

**Figure 2:**
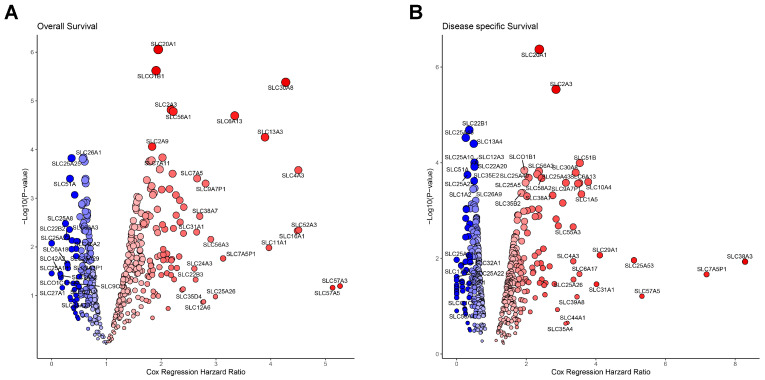
Prognostic significance of SLC genes in cancer. (**A**) Volcano plot showing the association between SLC gene expression and overall survival. (**B**) Volcano plot displaying the association between SLC gene expression and disease-specific survival. The x-axis represents the Cox regression hazard ratio, and the y-axis shows the –log10(*p*-value). Blue circles indicate SLC genes significantly associated with better survival, while red circles indicate those associated with worse survival.

Next, we evaluated the prognostic significance of all SLC genes in HNSC by conducting a Cox regression analysis on OS and DSS. The results are presented in Figure 2A,B, which depict the hazard ratios and *p*-values of SLC genes for OS and DSS, respectively, using a volcano plot format to visualize both the hazard ratio and –log10(*p*-value) for each SLC gene. The x-axis represents the Cox regression hazard ratio, and the y-axis shows the –log10(*p*-value). In the OS analysis, 239 SLC genes demonstrated statistical significance, with 155 associated with poor prognosis and 84 linked to favorable prognosis (Figure 2A). Similarly, the DSS analysis identified 268 SLC genes with statistical significance, where 145 were correlated with poor prognosis and 123 with favorable prognosis (Figure 2B). A total of 179 SLC genes exhibited statistically significant associations in both the OS and DSS analyses. Of these, 110 genes were associated with poor prognosis, while 69 genes were linked to favorable prognosis (Figure 2A,B). To more definitively correlate with poor prognosis, we selected 27 SLCs out of 110 that met all the cutoff finder criteria in terms of mean, distribution, and outcome significance.

### 3.3. Classification and Protein Levels of SLC Targets in HNSC

**Figure 3:**
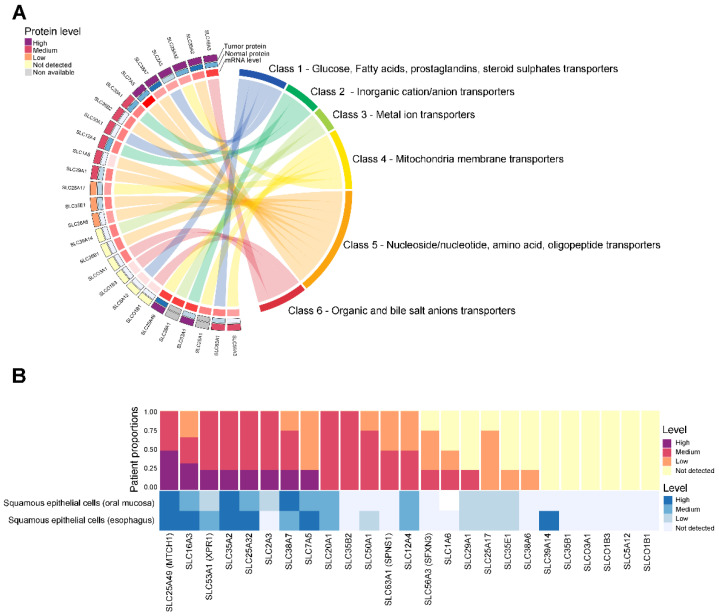
Classification and protein expression of SLC targets in HNSC. (**A**) The Chord diagram shows the protein levels detected in tumor (outer arc) and normal tissues (inner arc), with the expression levels indicated by color: high (dark purple), medium (red), low (orange), not detected (yellow), and non-available (grey). (**B**) The y-axis represents the patient proportions, while the x-axis lists the specific SLC proteins analyzed. The color bars represent the levels of protein expression detected in each cell type. Bar chart depicting the proportion of patients with high (dark purple), medium (red), low (orange), and not detected (yellow) expression levels of selected SLC proteins in HNSC. Bar chart depicting the protein level of SLC targets with high (dark blue), medium (blue), low (light blue), and not detected (very light blue) expression levels of selected SLC proteins in normal head and neck cells.

To identify significant targets for HNSC cancer, we selected 27 SLC genes that were consistently upregulated in tumor tissues compared with normal tissues and significantly associated with poor prognosis. Next, we conducted a detailed analysis of the classification and expression patterns of 27 SLC proteins to further elucidate their roles and significance in HNSC. The results are depicted in Figure 3. These SLC genes were categorized into eight major groups based on the types of solutes they transport, and their protein expression levels were examined (Figure 3A). In Figure 3A, 27 SLC genes are categorized into six classes based on the types of solutes they transport: Class 1 includes glucose, fatty acids, prostaglandins, and steroid sulfates transporters; Class 2 encompasses inorganic cation/anion transporters; Class 3 comprises metal ion transporters; Class 4 consists of mitochondrial membrane transporters; Class 5 includes nucleoside/nucleotide, amino acid, and oligopeptide transporters; and Class 6 covers organic and bile salt anion transporters. Among them, unfortunately, the protein levels for 2 SLC genes were non-available, and 6 SLC genes did not show detectable protein expression. Additionally, SLC genes from both Class 7, which includes transporters for urea, neurotransmitters, biogenic amines, ammonium, choline, and heme, as well as Class 8, encompassing vitamin and cofactor transporters, were not detected in this analysis.

To examine the expression patterns more closely, we analyzed the proportion of patients with varying expression levels of 25 selected SLC proteins in tumor versus normal tissues, as shown in Figure 3B. Notably, a high proportion and elevated expression levels were observed in the following proteins in HNSC: SLC16A3, SLC53A1, SLC25A32, SLC2A3, SLC38A7, and SLC7A5. However, despite the significant increase in the mRNA levels of some SLCs, the remaining 19 SLC proteins did not show a corresponding increase or high protein expression compared to normal levels.

### 3.4. Integrative Analysis of Selected 4 SLC Targets in HNSC

**Figure 4:**
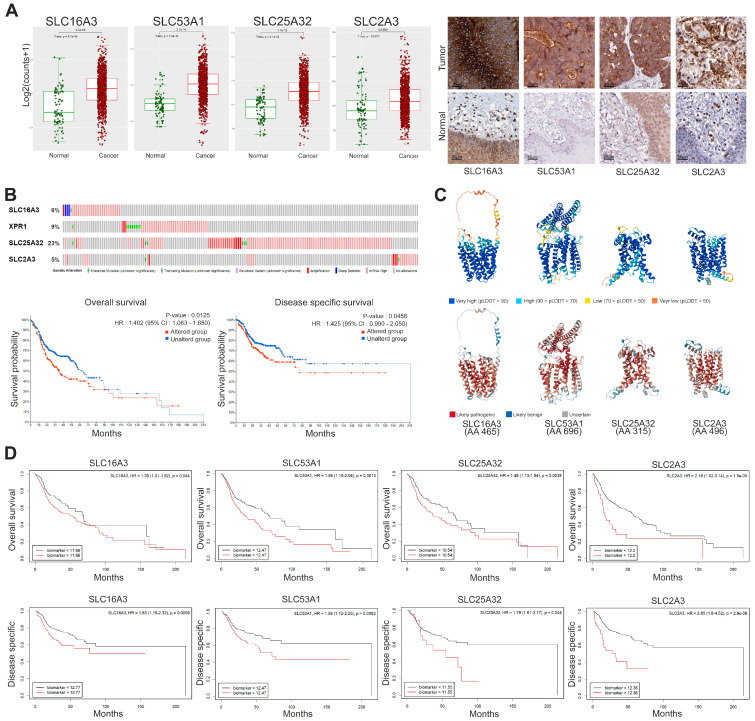
Expression, genetic alteration, and prognostic significance of selected SLC genes in cancer. (**A**) Box plots representing log2 expression levels of SLC16A3, SLC53A1, SLC25A32, and SLC2A3 in normal (green) versus cancer (red) tissues. Immunohistochemistry staining confirms high expression level and cell membrane localization in cancer tissues. Scale bars: 50 μm. (**B**) Genetic alteration frequencies of selected SLC genes. Kaplan-Meier plots showing overall survival and disease-specific survival for patients with altered (red) versus unaltered (blue) SLC genes. Statistical significance determined by log-rank test. (**C**) Predicted 3D structures of SLC proteins generated using AlphaFold2, color-coded by the predicted local distance difference test (pLDDT) scores: very high confidence (blue, pLDDT > 90), high confidence (light blue, 70 < pLDDT ≤ 90), low confidence (yellow, 50 < pLDDT ≤ 70), and very low confidence (red, pLDDT ≤ 50). Specific amino acid mutations were classified using AlphaMissense that categorizes missense mutations as likely pathogenic, likely benign, or uncertain based on evolutionary constraints and protein structure context. These mutations are mapped onto the structures, highlighting potential functional disruptions. (**D**) Kaplan-Meier survival curves illustrating the prognostic significance of high (red) versus low (black) expression levels of SLC genes for overall survival and disease-specific survival. Hazard ratios (HR) and *p*-values are provided.

To conduct a more integrative analysis, we selected SLC genes with high expression levels and a high proportion of patients exhibiting elevated levels. Specifically, we focused on SLC16A3, SLC53A1, SLC25A32, and SLC2A3 to investigate their expression levels and prognostic significance in HNSC. The results are presented in Figure 4, which includes multiple analyses such as differential expression, protein structure, genetic alteration, and survival outcomes. Figure 4A shows the significantly increased expression of SLC16A3, SLC53A1, SLC25A32, and SLC2A3 in cancer tissues compared to normal tissues. The immunohistochemistry results, depicted in the right panel of Figure 4A, further confirm the elevated protein levels of these SLCs in cancer tissues, providing visual evidence that supports the differential expression observed in the RNA-seq data. Additionally, these SLCs were localized on the cell membrane in HNSC, indicating their potential as promising therapeutic targets due to their accessible location.

Genetic alteration frequencies of the selected SLC genes reveal notable amplification, as shown in Figure 4B. Kaplan-Meier survival analyses indicate that genetic alterations in these SLC genes significantly correlate with worse overall survival (HR: 1.402, 95% CI: 1.063–1.850, *p* = 0.0125) and disease-specific survival (HR: 1.425, 95% CI: 0.990–2.050, *p* = 0.0458). These findings suggest a link between genetic alterations in SLC genes and adverse clinical outcomes. The predicted 3D structures of the SLC proteins, generated using AlphaFold2 and shown in Figure 4C, provide insights into their structural integrity and functional regions. The confidence levels of these predictions are indicated by the predicted local distance difference test (pLDDT) scores, with regions of very high confidence (pLDDT > 90) shown in blue and regions of very low confidence (pLDDT ≤ 50) in red. To assess the potential impact of missense mutations, we used AlphaMissense, an AI model that extends AlphaFold2 to categorize mutations as likely pathogenic, likely benign, or uncertain. This model leverages evolutionary constraints and structural context to estimate the pathogenicity of variants.

Kaplan-Meier survival curves (Figure 4D) demonstrate the prognostic significance of high versus low expression levels of SLC genes in both OS and DSS. High expression of SLC16A3 is associated with poorer overall survival (HR: 1.39, 95% CI: 1.01–1.92, *p* = 0.044) and disease-specific survival (HR: 1.63, 95% CI: 1.15–2.32, *p* = 0.0059). Similarly, SLC53A1 high expression levels are linked to worse outcomes, with an overall survival (HR: 1.56, 95% CI: 1.19–2.04, *p* = 0.0012) and disease-specific survival (HR: 1.58 95% CI: 1.12–2.25, *p* = 0.0093). SLC25A32 high expression correlates with poorer overall survival (HR: 1.48, 95% CI: 1.13–1.94, *p* = 0.0038) and disease-specific survival (HR: 1.79, 95% CI: 1.01–3.17, *p* = 0.044). Lastly, SLC2A3 high expression is associated with worse overall survival (HR: 2.18, 95% CI: 1.52–3.14, *p* = 1.5 × 10^−5^) and disease-specific survival (HR: 2.85, 95% CI: 1.8–4.52, *p* = 2.9 × 10^−6^).

### 3.5. Gene Set Enrichment Analysis (GSEA) of 4 SLC Targets in HNSC

**Figure 5:**
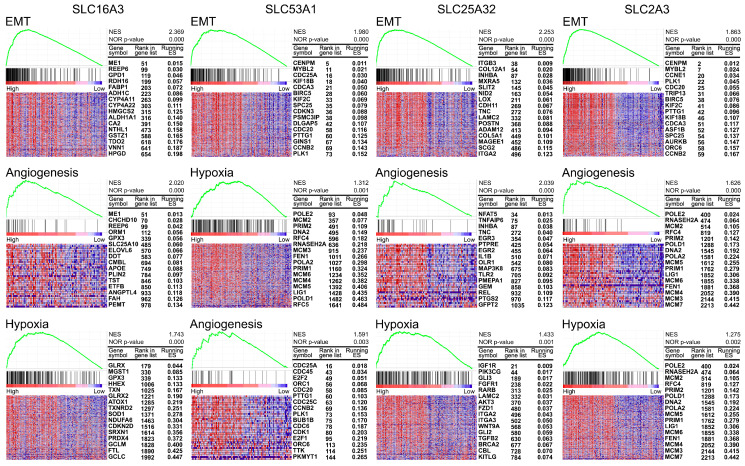
Gene Set Enrichment Analysis (GSEA) of selected SLC targets in HNSC. Heatmaps and enrichment plots of pathways significantly enriched in cancers with high expression of SLC16A3, SLC53A1, SLC25A32, and SLC2A3. Each panel shows the top pathways with their corresponding normalized enrichment scores (NES) and nominal *p*-values. The heatmaps illustrate the expression levels of key genes within each pathway, with samples ordered by their expression levels of the respective SLC gene. For each gene, the rank in the gene list and the corresponding running enrichment score (ES) are provided.

To understand the mechanisms associated with the selected four SLCs, we performed GSEA to identify pathways enriched in cancers with high expression of SLC16A3, SLC53A1, SLC25A32, and SLC2A3. The results, illustrated in Figure 5, highlight significant enrichment in several oncogenic pathways. For SLC16A3, notable enrichment was observed in the epithelial-mesenchymal transition (EMT) pathway (NES = 2.369, *p*-value = 0.000), angiogenesis pathway (NES = 2.020, *p*-value = 0.000), and hypoxia pathway (NES = 1.743, *p*-value = 0.000). SLC53A1 showed significant enrichment in the EMT pathway (NES = 1.980, *p*-value = 0.000), hypoxia pathway (NES = 1.312, *p*-value = 0.001), and angiogenesis pathway (NES = 1.591, *p*-value = 0.003). For SLC25A32, significant enrichment was detected in the EMT pathway (NES = 2.253, *p*-value = 0.000), angiogenesis pathway (NES = 2.039, *p*-value = 0.000), and hypoxia pathway (NES = 1.433, *p*-value = 0.001). SLC2A3 was associated with enrichment in multiple pathways, including EMT (NES = 1.863, *p*-value = 0.000), angiogenesis (NES = 1.626, *p*-value = 0.000), and hypoxia (NES = 1.275, *p*-value = 0.002).

## 4. Discussion

Precision oncology leverages cutting-edge genomic technologies to characterize diseases at an unprecedented level of detail, enabling the development of treatments tailored to individual genetic profiles and the specific characteristics of tumors [13]. A critical aspect of this approach involves the solute carrier (SLC) transporter superfamily, which includes over 400 members across 65 families [10]. These transporters are integral to the movement of ions, metabolites, and drugs across cellular membranes. Notably, many SLC transporters are expressed on the cell surface, making them accessible targets for small molecule therapies in cancer treatment [20]. In the context of cancer, SLC transporters are particularly important as they fulfill the elevated metabolic needs of tumor cells by facilitating the transport of essential nutrients, including amino acids, nucleotides, and other key metabolites [11]. The functional diversity of SLC transporters across different cancer types underscores the necessity of a tumor-specific understanding of these proteins. Such insights are vital for the identification of new therapeutic targets and the development of next-generation SLC-based cancer treatments. Therefore, continued research into the role of SLC transporters in oncology is imperative for advancing precision medicine and improving patient outcomes.

This study emphasizes the pivotal roles of SLC transporters, specifically SLC16A3, SLC53A1, SLC25A32, and SLC2A3, in the pathophysiology of HNSC, highlighting their potential as therapeutic targets. Through the integration of mRNA and protein expression data from TCGA and HPA, our comprehensive analysis revealed a significant upregulation of these SLC genes in HNSC compared to normal tissues, correlating with a poorer prognosis. GSEA revealed significant enrichment of several oncogenic pathways, including EMT, angiogenesis, and hypoxia signaling, in cancers with elevated expression of these SLC transporters. These pathways are well-known drivers of cancer progression, metastasis, and changes in the tumor microenvironment. The EMT pathway, in particular, is linked to increased migratory and invasive properties of cancer cells, thereby promoting metastasis [21]. The enrichment of angiogenesis and hypoxia pathways further underscores the involvement of these SLC transporters in sustaining the aggressive behavior of HNSC [22].

In normal head and neck tissues, the most highly expressed SLCs, in order, were SLC42A3, SLC25A6, and SLC25A3 among the 446 SLC genes. The SLC42 family, also known as the Rh ammonium transporter family, includes Rh glycoproteins which are involved in acid-base transport and potentially in the transport of gases such as CO2 and NH3, with significant roles in various tissues [23]. Despite the high expression of SLC42A3 in normal head and neck tissues, its function remains poorly understood. The SLC25 family is classified as the mitochondrial carrier family, encompassing a variety of transporters that facilitate the exchange of metabolites across the inner mitochondrial membrane [24]. SLC25A6, also known as adenine nucleotide translocase-3 (ANT3), functions as an exchanger, specifically transporting ADP into the mitochondria and ATP out of it, which is crucial for maintaining cellular energy balance [25]. Similarly, SLC25A3, known as the phosphate carrier (PHC), plays a key role in transporting inorganic phosphate into the mitochondria, a process essential for ATP synthesis and overall mitochondrial function [25]. Both of these transporters are integral to the efficient operation of cellular metabolism and energy production in normal head and neck tissues [24,25].

In our study, the persistent overexpression of SLC16A3, SLC53A1, SLC25A32, and SLC2A3 at both the mRNA and protein levels in malignant tissues was strongly correlated with a poorer prognosis in HNSC. The SLC16 family, comprising 14 solute carrier proteins and commonly referred to as the monocarboxylate transporter (MCT) family, is essential for the transmembrane transport of monocarboxylates such as lactate, pyruvate, and ketone bodies [26]. Glycolytic metabolism, driven by the Warburg effect, necessitates the continuous export of lactate from cells via MCTs, contributing to the accumulation of lactic acid within the tumor microenvironment [27]. Targeting the MCT4 lactate transporter offers the potential to sequester lactate within cancer cells, disrupting their metabolic processes and reducing lactate levels in the tumor microenvironment, which may help reverse immune suppression [28]. In our study, we observed a significant upregulation of SLC16A3, also known as MCT4, at both the mRNA and protein levels in HNSC patients. This elevated expression was strongly associated with poorer prognosis, indicating that SLC16A3 could serve as a valuable prognostic biomarker and a potential therapeutic target in HNSC. Moreover, GSEA revealed that SLC16A3 is significantly linked to the EMT, angiogenesis, and hypoxia pathways. Ongoing research into MCT4 inhibitors is advancing [29], and our findings suggest that these inhibitors could have therapeutic implications for the treatment of HNSC.

The SLC53A1 gene, which encodes the xenotropic and polytropic retrovirus receptor 1 (XPR1), is an integral member of the solute carrier family, specifically involved in the regulation of phosphate export from cells [30]. XPR1 plays a vital role in maintaining cellular phosphate homeostasis, a process crucial for a wide range of biological functions, including nucleotide synthesis, energy metabolism, and intracellular signaling pathways [31,32]. The proper balance of phosphate within cells is essential for sustaining cellular functions and viability, and disturbances in phosphate transport mechanisms are increasingly recognized as contributing factors to tumor development and progression [33,34]. In our study, we observed a significant overexpression of SLC53A1 at both the mRNA and protein levels in HNSC tissues compared to normal tissues. This overexpression was strongly correlated with a poorer clinical prognosis, underscoring the potential of SLC53A1 as a valuable prognostic biomarker and potential therapeutic targets in HNSC. Moreover, GSEA revealed that SLC53A1 is also significantly linked to the EMT, angiogenesis, and hypoxia pathways. Our findings suggest that targeting XPR1 could represent a novel therapeutic approach for treating HNSC, offering the possibility of disrupting critical phosphate pathways that sustain tumor growth and progression. Given the emerging understanding of phosphate transport in cancer biology, it is imperative that research into the development of XPR1 inhibitors rapidly advances.

Our study also revealed a significant upregulation of SLC25A32, the gene encoding the mitochondrial folate transporter (MTF), at both the mRNA and protein levels in HNSC. SLC25A32 is essential for the transport of folate into mitochondria, a process crucial for maintaining mitochondrial function and one-carbon metabolism, which are key to nucleotide synthesis and cellular proliferation [35,36]. We also found that SLC2A3, which encodes the glucose transporter protein GLUT3, was upregulated. GLUT3 is primarily responsible for high-affinity glucose uptake, playing a critical role in meeting the increased metabolic demands of rapidly proliferating cells [37]. This elevated expression was strongly associated with poorer prognosis, indicating that SLC25A32 and SLC2A3 could serve as valuable prognostic biomarkers and potential therapeutic targets in HNSC. Moreover, GSEA revealed that both SLC25A32 and SLC2A3 are significantly linked to the EMT, angiogenesis, and hypoxia pathways. The upregulation of these genes in HNSC suggests a metabolic adaptation within cancer cells, where enhanced mitochondrial folate transport and glucose uptake may be necessary to support not only the rapid growth but also their ability to invade, induce new blood vessel formation, and thrive in low-oxygen environments [38,39,40].

While our study offers significant insights, it is important to recognize certain limitations. The use of publicly accessible databases may have introduced potential biases due to differences in sample collection, processing methods, and data annotation. Furthermore, the identified SLC targets require functional validation in experimental models to substantiate their roles in HNSC and their viability as therapeutic targets. To advance this field, future research should focus on a detailed investigation of the mechanistic roles of these transporters in HNSC, utilizing both in vitro and in vivo models. This approach will be crucial in determining their potential impact on therapeutic strategies.

## 5. Conclusions

Our study provides an in-depth investigation of the solute carrier (SLC) transporter superfamily in HNSC, highlighting their potential as both prognostic biomarkers and therapeutic targets. Through the integration of multi-omics data with advanced computational methodologies, we have identified specific SLC transporters—SLC16A3, SLC53A1, SLC25A32, and SLC2A3—as key players in the progression of HNSC. These findings suggest that targeting these transporters with small molecule inhibitors could disrupt essential metabolic pathways in cancer cells, offering a novel therapeutic approach to counteract this aggressive malignancy. Our work lays a critical foundation for the development of SLC-based target therapies in precision oncology, aiming to enhance treatment outcomes for patients afflicted by this challenging disease.

## Data Availability

All the data included in this study are available, including TCGA-HNSC (https://xenabrowser.net/datapages/?cohort=GDC TCGA Head and Neck Cancer (HNSC), accessed on 5 January 2024), HPA (http://www.proteinatlas.org, accessed on 12 January 2024), and cBioPortal of Cancer Genomics (http://www.cbioportal.org/, accessed on 5 May 2024).

## Supplementary Materials

The following supporting information can be downloaded at: https://www.mdpi.com/article/10.3390/cancers16183226/s1, Table S1: Summary of 446 SLC Transporters.
