# Supplementary material for: The Solute Carrier (SLC) Transporter Superfamily as Therapeutic Targets for the Treatment of Head and Neck Squamous Cell Carcinoma
Source: Cancers (Basel). 2024 Sep 22;16(18):3226. doi: 10.3390/cancers16183226 (PMC11430461; doi:10.3390/cancers16183226)
Supplement: Supplementary file 1 [file cancers-16-03226-s001.zip › cancers-3203098-supplementary.pdf]

**Supplementary Table S1.** Summary of 446 SLC Transporters

| No. | SLC name | Ensembl ID | Protein name | Aliases | Transport type* | Substrates |
|---|---|---|---|---|---|---|
| 1 | SLC1A1 | ENSG00000106688.11 | EAAC1, EAAT3 | System X-AG | C / Na+, H+, K+ | L-Glu, D/L-Asp |
| 2 | SLC1A2 | ENSG00000110436.11 | GLT-1, EAAT2 | System X-AG | C / Na+, H+, K+ | L-Glu, D/L-Asp |
| 3 | SLC1A3 | ENSG00000079215.13 | GLAST, EAAT1 | System X-AG | C / Na+, H+, K+ | L-Glu, D/L-Asp |
| 4 | SLC1A4 | ENSG00000115902.10 | ASCT1, SATT | System ASC | C / Na+, E / amino acids | L-Ala, L-Ser, L-Cys, L-Thr |
| 5 | SLC1A5 | ENSG00000105281.12 | ASCT2, AAAT | System ASC | C / Na+, E / amino acids | L-Ala, L-Ser, L-Cys, L-Thr, L-Gln, L-Asn |
| 6 | SLC1A6 | ENSG00000105143.12 | EAAT4 | System X-AG | C / Na+, H+, K+ | L-Glu, D/L-Asp |
| 7 | SLC1A7 | ENSG00000162383.11 | EAAT5 | System X-AG | C / Na+, H+, K+ | L-Glu, D/L-Asp |
| 8 | SLC2A1 | ENSG00000117394.19 | GLUT1 | | F | glucose, galactose, mannose, glucosamine |
| 9 | SLC2A2 | ENSG00000163581.13 | GLUT2 | | F | glucose, galactose, fructose, mannose, glucosamine |
| 10 | SLC2A3 | ENSG00000059804.15 | GLUT3 | | F | glucose, galactose, mannose, xylose |
| 11 | SLC2A3P1 | ENSG00000253861.1 | pseudogene | GLUT3 pseudogene 1 | | |
| 12 | SLC2A3P2 | ENSG00000185031.6 | pseudogene | GLUT3 pseudogene 2 | | |
| 13 | SLC2A3P4 | ENSG00000254088.1 | pseudogene | GLUT3 pseudogene 4 | | |
| 14 | SLC2A4 | ENSG00000181856.14 | GLUT4 | | F | glucose, glucosamine |
| 15 | SLC2A5 | ENSG00000142583.17 | GLUT5 | | F | fructose |
| 16 | SLC2A6 | ENSG00000160326.13 | GLUT6 | GLUT9 | F | glucose |
| 17 | SLC2A7 | ENSG00000197241.3 | GLUT7 | | F | glucose, fructose |
| 18 | SLC2A8 | ENSG00000136856.17 | GLUT8 | GLUTX1 | F | glucose, fructose, galactose |
| 19 | SLC2A9 | ENSG00000109667.11 | GLUT9 | GLUTX, URATv1 | | urate (glucose, fructose) |
| 20 | SLC2A10 | ENSG00000197496.5 | GLUT10 | | F | glucose, galactose |
| 21 | SLC2A11 | ENSG00000133460.19 | GLUT11 | GLUT10 | F | glucose, fructose |
| 22 | SLC2A12 | ENSG00000146411.5 | GLUT12 | GLUT8 | F | glucose |
| 23 | SLC2A13 | ENSG00000151229.12 | HMIT | GLUT13 | C / H+ | myo-inositol |
| 24 | SLC2A14 | ENSG00000173262.11 | GLUT14 | SLC2A3P3 | O | |
| 25 | SLC3A1 | ENSG00000138079.13 | rBAT | NBAT, D2H | E (see details in SLC7 table) | system b0,+, heterodimerizes with light subunit SLC7A9 |
| 26 | SLC3A2 | ENSG00000168003.16 | 4F2hc | CD98hc, FRP | E (see details in SLC7 table) | systems L, y+L, xc- and asc with light subunits SLC7A5-8 and SLC7A10-11 |
| 27 | SLC4A1 | ENSG00000004939.13 | AE1 | Band 3 | E | chloride cicarbonate |

| | | | | | | |
|---|---|---|---|---|---|---|
| 28 | SLC4A2 | ENSG00000164889.12 | AE2 | | E | chloride bicarbonate |
| 29 | SLC4A3 | ENSG00000114923.16 | AE3 | | E | chloride bicarbonate |
| 30 | SLC4A4 | ENSG00000080493.13 | NBCe1 | NBC, NBC1 | C | sodium bicarbonate (and/or carbonate) |
| 31 | SLC4A5 | ENSG00000188687.15 | NBCe2 | NBC4 | C | sodium bicarbonate (and/or carbonate) |
| 32 | SLC4A7 | ENSG00000033867.16 | NBCn1 | NBC2, NBC3, SLC4A6 | C | chloride bicarbonate |
| 33 | SLC4A8 | ENSG00000050438.16 | NDCBE | kNBC3 | C, E | sodium bicarbonate chloride |
| 34 | SLC4A9 | ENSG00000113073.14 | | AE4 | | inconclusive |
| 35 | SLC4A10 | ENSG00000144290.16 | NBCn2 | NCBE | C, E | sodium bicarbonate chloride |
| 36 | SLC4A11 | ENSG00000088836.12 | BTR1 | NaBC1 | C | sodium, borate |
| 37 | SLC5A1 | ENSG00000100170.9 | SGLT1 | None | C / Na+, (H+), F / Na+, (H+), Ch / urea, water | glucose and galactose (urea and water) |
| 38 | SLC5A2 | ENSG00000140675.12 | SGLT2 | None | C / Na+ | glucose |
| 39 | SLC5A3 | ENSG00000198743.5 | SMIT1 | None | C / Na+ | myoinositol (glucose) |
| 40 | SLC5A4 | ENSG00000100191.5 | SGLT3 | SAAT1 | glucose activated Na+, (H+) channel | Na+ (H+) |
| 41 | SLC5A5 | ENSG00000105641.3 | NIS | | C / Na+, F / Na+, Ch / urea, water | I- (ClO4-, SCN-, NO3-, Br-) |
| 42 | SLC5A6 | ENSG00000138074.14 | SMVT | | C / Na+ | biotin, lipoate panthothenate, I- |
| 43 | SLC5A7 | ENSG00000115665.8 | CHT | CHT1 | | choline |
| 44 | SLC5A8 | ENSG00000256870.2 | SMCT1 | AIT | C / Na+ | short chain fatty acids |
| 45 | SLC5A9 | ENSG00000117834.12 | SGLT4 | | C / Na+ | mannose, fructose, glucose |
| 46 | SLC5A10 | ENSG00000154025.15 | SGLT5 | RK-D | C / Na+ | mannose, fructose, glucose |
| 47 | SLC5A11 | ENSG00000158865.12 | SMIT2 | SGLT6, KST1 | C / Na+ | myoinositol, chiro-inositol |
| 48 | SLC5A12 | ENSG00000148942.14 | SMCT2 | | C / Na+ | short chain fatty acids |
| 49 | SLC6A1 | ENSG00000157103.10 | | GAT1 | | GABA |
| 50 | SLC6A2 | ENSG00000103546.18 | | NAT1, NET1, NET | | norepinephrine |
| 51 | SLC6A3 | ENSG00000142319.17 | | DAT, DAT1 | | dopamine |
| 52 | SLC6A4 | ENSG00000108576.9 | | 5-HTT, SERT | | serotonin |
| 53 | SLC6A5 | ENSG00000165970.11 | | GlyT2 | | glycine |
| 54 | SLC6A6 | ENSG00000131389.16 | | TauT | | taurine |
| 55 | SLC6A7 | ENSG00000011083.8 | | PROT | | proline |

| | | | | | | |
|---|---|---|---|---|---|---|
| 56 | SLC6A8 | ENSG00000130821.15 | | CRTR, CT1 | | creatine |
| 57 | SLC6A9 | ENSG00000196517.11 | | GlyT1 | | glycine |
| 58 | SLC6A10P | ENSG00000214617.9 | pseudogene | CT2 | | |
| 59 | SLC6A11 | ENSG00000132164.9 | | GAT-B, GAT-3 | | GABA |
| 60 | SLC6A12 | ENSG00000111181.12 | | BGT1 | | betaine, GABA |
| 61 | SLC6A13 | ENSG00000010379.15 | | GAT2 | | GABA |
| 62 | SLC6A14 | ENSG00000268104.2 | | ATB0+, beta-alanine carrier system | | neutral, cationic amino acids |
| 63 | SLC6A15 | ENSG00000072041.16 | | B0AT2, v7-3, NTT7-3 | | large, neutral amino acids |
| 64 | SLC6A16 | ENSG00000063127.15 | | NTT5 | | unknown |
| 65 | SLC6A17 | ENSG00000197106.6 | | NTT4, XT1 | | neutral amino acids |
| 66 | SLC6A18 | ENSG00000164363.9 | | B0AT3, XT2 | | neutral amino acids |
| 67 | SLC6A19 | ENSG00000174358.15 | | B0AT1, HND | | neutral amino acids |
| 68 | SLC6A20 | ENSG00000163817.15 | | system IMINO, XT3, Xtrp3 | | proline, pipecolate, sarcosine |
| 69 | SLC7A1 | ENSG00000139514.12 | CAT-1 | ATRC1, system y+ | F (non-obligatory E) | cationic L-amino acids |
| 70 | SLC7A2 | ENSG00000003989.16 | CAT-2 (A or B) | ATRC2, system y+ | F | cationic L-amino acids |
| 71 | SLC7A3 | ENSG00000165349.11 | CAT-3 | ATRC3, system y+ | F | cationic L-amino acids |
| 72 | SLC7A4 | ENSG00000099960.12 | CAT-4 | | O | |
| 73 | SLC7A5 | ENSG00000103257.8 | LAT1 | [4F2hc], 4F2lc, system L | E (similar intra- and extracellular selectivities, lower intracellular apparent affinity) | large neutral L-amino acids, T3, T4, L-DOPA, BCH |
| 74 | SLC7A5P1 | ENSG00000260727.1 | pseudogene | LAT1-3TM, MLAS | | |
| 75 | SLC7A6 | ENSG00000103064.13 | y+LAT2 | [4F2hc], system y+L | E (preferentially intracellular cationic amino acid against extracellular neutral amino acid / Na+) | cationic amino acids (Na+ indep.), large neutral L-amino acids (Na+ dep.) |
| 76 | SLC7A7 | ENSG00000155465.18 | y+LAT1 | [4F2hc], system y+L | E (preferentially intracellular cationic amino acid against extracellular neutral amino acid / Na+) | cationic amino acids (Na+ indep.), large neutral L-amino acids (Na+ dep.) |

| | | | | | | |
|---|---|---|---|---|---|---|
| 77 | SLC7A8 | ENSG00000092068.18 | LAT2 | [4F2hc], system L | E (similar intra- and extracellular selectivities, lower intracellular apparent affinity) | neutral L-amino acids, T3, T4, BCH |
| 78 | SLC7A9 | ENSG00000021488.12 | b0,+AT | [rBAT], system b0,+ | E (preferentially extracellular cationic amino acid and cystine against intracellular neutral amino acid) | cationic amino acids, large neutral amino acids |
| 79 | SLC7A10 | ENSG00000130876.11 | Asc-1 | [4F2hc], system asc | preferentially E | small neutral amino acids |
| 80 | SLC7A11 | ENSG00000151012.13 | xCT | [4F2hc], system xc- | E (preferentially extracellular cystine against intracellular glutamate) | cystine (anionic form), L-glutamate |
| 81 | SLC7A13 | ENSG00000164893.8 | AGT-1 | XAT2 | E | L-aspartate, L-glutamate |
| 82 | SLC7A14 | ENSG00000013293.5 | | | O | |
| 83 | SLC7A15P | ENSG00000232800.1 | pseudogene | | | |
| 84 | SLC8A1 | ENSG00000183023.18 | NCX1 | NACA, NCE | E / Na+, Ca2+ | Na+, Ca2+ |
| 85 | SLC8A2 | ENSG00000118160.13 | NCX2 | | E / Na+, Ca2+ | Na+, Ca2+ |
| 86 | SLC8A3 | ENSG00000100678.18 | NCX3 | | E / Na+, Ca2+ | Na+, Ca2+ |
| 87 | SLC8B1 | ENSG00000089060.11 | NCLX | mNCX | E / Na+, Li+, Ca2+ | Na+, Li+, Ca2+ |
| 88 | SLC9A1 | ENSG00000090020.10 | NHE1 | APNH? | E / Na+, H+ | Na+, Li+, H+, NH4+ |
| 89 | SLC9A2 | ENSG00000115616.2 | NHE2 | | E / Na+, H+ | Na+, Li+, H+, NH4+ |
| 90 | SLC9A3 | ENSG00000066230.10 | NHE3 | | E / Na+, H+ | Na+, Li+, H+, NH4+ |
| 91 | SLC9A3P1 | ENSG00000233011.1 | pseudogene | | | |
| 92 | SLC9A3P2 | ENSG00000238125.1 | pseudogene | | | |
| 93 | SLC9A3P3 | ENSG00000226631.1 | pseudogene | | | |
| 94 | SLC9A4 | ENSG00000180251.4 | NHE4 | | E / Na+, H+ | Na+, Li+ (?), H+, NH4+ |
| 95 | SLC9A5 | ENSG00000135740.16 | NHE5 | | E / Na+, H+ | Na+, Li+, H+, NH4+ (?) |
| 96 | SLC9A6 | ENSG00000198689.9 | NHE6 | | E / Na+ (K+), H+ | Na+, K+, H+ |
| 97 | SLC9A7 | ENSG00000065923.9 | NHE7 | | E / Na+ (K+), H+ | Na+, K+, Li+, H+, NH4+ (?) |
| 98 | SLC9A7P1 | ENSG00000227825.4 | pseudogene, partially processed | | | |
| 99 | SLC9A8 | ENSG00000197818.11 | NHE8 | | E / Na+ (K+), H+ | Na+, K+, H+ |

| | | | | | | |
|---|---|---|---|---|---|---|
| 100 | SLC9A9 | ENSG00000181804.14 | NHE9 | | E / Na+ (K+), H+ | Na+, K+, H+ |
| 101 | SLC9B1 | ENSG00000164037.16 | NHA1 | NHEDC1 | | |
| 102 | SLC9B2 | ENSG00000164038.14 | NHA2 | NHEDC2 | | Na+, Li+ |
| 103 | SLC9C1 | ENSG00000172139.14 | Sperm-NHE | | | Na+, H+ |
| 104 | SLC9C2 | ENSG00000162753.11 | | | | |
| 105 | SLC10A1 | ENSG00000100652.4 | NTCP | LBAT | C / Na+ | bile acids |
| 106 | SLC10A2 | ENSG00000125255.6 | ASBT | IBAT, ISBT, NTCP2 | C / Na+ | bile acids |
| 107 | SLC10A3 | ENSG00000126903.15 | P3 | | O | |
| 108 | SLC10A4 | ENSG00000145248.6 | P4 | | O | |
| 109 | SLC10A5 | ENSG00000253598.1 | P5 | | O | |
| 110 | SLC10A6 | ENSG00000145283.7 | SOAT | P6 | C / Na+ | estrone-3-sulfate, dehydroepiandrosterone sulfate, pregnenolone sulfate |
| 111 | SLC10A7 | ENSG00000120519.14 | P7 | C4orf13 | O | |
| 112 | SLC11A1 | ENSG00000018280.16 | NRAMP1 | NRAMP, LSH | E or C ? / H+ | Mn2+, Fe2+, other divalent metal ions |
| 113 | SLC11A2 | ENSG00000110911.14 | DMT1 | NRAMP2, DCT1 | C / H+ | Fe2+, Cd2+, Co2+, Cu1+, Mn2+, Ni2+, Pb2+, Zn2+ |
| 114 | SLC12A1 | ENSG00000074803.17 | NKCC2 | | | Na+, K+, Cl- |
| 115 | SLC12A2 | ENSG00000064651.13 | NKCC1 | | | Na+, K+, Cl- |
| 116 | SLC12A3 | ENSG00000070915.9 | NCC | | | Na+, Cl- |
| 117 | SLC12A4 | ENSG00000124067.16 | KCC1 | | | K+, Cl- |
| 118 | SLC12A5 | ENSG00000124140.12 | KCC2 | | | K+, Cl- |
| 119 | SLC12A6 | ENSG00000140199.11 | KCC3 | | | K+, Cl- |
| 120 | SLC12A7 | ENSG00000113504.19 | KCC4 | | | K+, Cl- |
| 121 | SLC12A8 | ENSG00000221955.10 | CCC9 | | | unknown |
| 122 | SLC12A9 | ENSG00000146828.17 | CIP | | | polyamines? |
| 123 | SLC13A1 | ENSG00000081800.8 | NaS1 | Na-sulfate, NaSi-1 | C / Na+ | sulfate, selenate, thiosulfate |
| 124 | SLC13A2 | ENSG00000007216.14 | NaC1 | SDCT1, NaDC-2, NaDC1 | C / Na+ | succinate, citrate, ?-ketoglutarate |
| 125 | SLC13A3 | ENSG00000158296.13 | NaC3 | NaDC3, NaC3, SDCT2 | C / Na+ | succinate, citrate, ?-ketoglutarate, NALA, glutarate and its derivatives |
| 126 | SLC13A4 | ENSG00000164707.15 | NaS2 | SUT1 | C / Na+ | sulfate, oxyanions selenium and chromium |
| 127 | SLC13A5 | ENSG00000141485.15 | NaC2 | NaCT | C / Na+ | citrate, succinate, pyruvate |

| | | | | | | |
|---|---|---|---|---|---|---|
| 128 | SLC14A1 | ENSG00000141469.16 | | UT3, UT11, JK, Kidd antigen | | |
| 129 | SLC14A2 | ENSG00000132874.13 | | UT1 | | |
| 130 | SLC15A1 | ENSG00000088386.15 | PEPT1 | oligopeptide transporter 1, H+-peptide transporter 1 | C / H+ | di- and tri-peptides, protons, beta-lactam antibiotics |
| 131 | SLC15A2 | ENSG00000163406.10 | PEPT2 | oligopeptide transporter 2, H+-peptide transporter 2 | C / H+ | di- and tri-peptides, protons, beta-lactam antibiotics |
| 132 | SLC15A3 | ENSG00000110446.9 | PHT2 | peptide/histidine transporter 2, PTR3 | C / H+ | di- and tri-peptides, protons, histidine |
| 133 | SLC15A4 | ENSG00000139370.10 | PHT1 | peptide/histidine transporter 1, PTR4 | C / H+ | di- and tri-peptides, protons, histidine |
| 134 | SLC16A1 | ENSG00000155380.11 | MCT1, | MOT1 | C / H+ or E / monocarboxylate | lactate, pyruvate, ketone bodies |
| 135 | SLC16A2 | ENSG00000147100.9 | MCT8 | MOT8, XPCT, MCT7 | F | T2, rT3, T3, T4 |
| 136 | SLC16A3 | ENSG00000141526.14 | MCT4 | MOT4, MCT3 | C / H+ | lactate, ketone bodies |
| 137 | SLC16A4 | ENSG00000168679.17 | MCT5 | MOT5, MCT4 | O | |
| 138 | SLC16A5 | ENSG00000170190.15 | MCT6 | MOT6, MCT5 | | bumetanide, probenecid, nateglinide ? |
| 139 | SLC16A6 | ENSG00000108932.11 | MCT7 | MOT7, MCT6 | O | |
| 140 | SLC16A7 | ENSG00000118596.11 | MCT2 | MOT2 | C / H+ | pyruvate, lactate, ketone bodies |
| 141 | SLC16A8 | ENSG00000100156.10 | MCT3 | MOT3, REMP | C / H+ (pH dependent but cotransport not confirmed experimentally) | lactate |
| 142 | SLC16A9 | ENSG00000165449.11 | MCT9 | MOT9 | O | |
| 143 | SLC16A10 | ENSG00000112394.16 | TAT1, MCT10 | MOT10 | F | aromatic amino acids, T3, T4 |
| 144 | SLC16A11 | ENSG00000174326.11 | MCT11 | MOT11 | O | |
| 145 | SLC16A12 | ENSG00000152779.13 | MCT12 | MOT12 | O | |
| 146 | SLC16A13 | ENSG00000174327.6 | MCT13 | MOT13 | O | |
| 147 | SLC16A14 | ENSG00000163053.10 | MCT14 | MOT14 | O | |
| 148 | SLC17A1 | ENSG00000124568.10 | NPT1 | NaPi1 | electrogenic, Cl-dependent; C/Na+; channel | organic anions, phosphate, chloride |

| | | | | | | |
|---|---|---|---|---|---|---|
| 149 | SLC17A2 | ENSG00000112337.10 | NPT3 | | unknown | unknown |
| 150 | SLC17A3 | ENSG00000124564.17 | NPT4 | | electrogenic | organic anions |
| 151 | SLC17A4 | ENSG00000146039.10 | Na+/PO4-cotransporter homologue | | unknown | unknown |
| 152 | SLC17A5 | ENSG00000119899.12 | sialin | AST, VEAT | C / H+ | sialic acid, other acidic sugars |
| 153 | SLC17A6 | ENSG00000091664.7 | VGLUT2 | DNPI | electrogenic, Cl-dependent | glutamate |
| 154 | SLC17A7 | ENSG00000104888.9 | VGLUT1 | BNPI | electrogenic, Cl-dependent | glutamate |
| 155 | SLC17A8 | ENSG00000179520.10 | VGLUT3 | | electrogenic, Cl-dependent | glutamate |
| 156 | SLC17A9 | ENSG00000101194.17 | VNUT | | electrogenic, Cl-dependent | purine nucleotides |
| 157 | SLC18A1 | ENSG00000036565.14 | VMAT1 | CGAT, VAT | E / H+ | 5-HT, DA, NE, epinephrine |
| 158 | SLC18A2 | ENSG00000165646.11 | VMAT2 | SVAT, SVMT, VAT2, MAT | E / H+ | 5-HT, DA, NE, epinephrine, histamine |
| 159 | SLC18A3 | ENSG00000187714.6 | VAChT | Unc-17, Vesamicol binding protein | E / H+ | acetylcholine |
| 160 | SLC18B1 | ENSG00000146409.10 | C6orf192 | hypothetical human protein LOC116843, dJ55C23.6 gene product | unknown | unknown |
| 161 | SLC19A1 | ENSG00000173638.18 | RFC | RFC1, RFT | E / organic phosphates | reduced folates, antifolates |
| 162 | SLC19A2 | ENSG00000117479.12 | THTR1 | ThTr1 | F | thiamine |
| 163 | SLC19A3 | ENSG00000135917.13 | THTR2 | ThTr2 | F | thiamine |
| 164 | SLC20A1 | ENSG00000144136.10 | PiT-1 | gibbon ape leukemia virus receptor 1, GLVR1, Glvr1, FLJ41426, DKFZp686J2397 | C / Na+, H2PO4- | inorganic phosphate (monovalent) |
| 165 | SLC20A2 | ENSG00000168575.9 | PiT-2 | amphotropic murine leukemia virus receptor 2, GLVR2, Glvr-2, MLVAR | C / Na+, H2PO4- | inorganic phosphate (monovalent) |
| 166 | SLCO2A1 | ENSG00000174640.12 | OATP2A1 | | | prostaglandins (C/lactate) |

| | | | | | | |
|---|---|---|---|---|---|---|
| 167 | SLCO4A1 | ENSG00000101187.15 | | OATP-E, OATP4A1 | | |
| 168 | SLCO5A1 | ENSG00000137571.10 | | OATPRP4, OATP-J, OATP5A1 | | |
| 169 | SLCO3A1 | ENSG00000176463.13 | | OATP-D, OATP3A1 | | |
| 170 | SLCO6A1 | ENSG00000205359.9 | | OATP6A1, OATPY, MGC26949, CT48 | | |
| 171 | SLCO1A2 | ENSG00000084453.16 | OATP1A2 | | | bile salts, organic anions and cations |
| 172 | SLCO2B1 | ENSG00000137491.14 | OATP2B1 | | | E-3-S, DHEAS |
| 173 | SLCO1B1 | ENSG00000134538.2 | OATP1B1 | | | bile salts, organic anions |
| 174 | SLCO1B3 | ENSG00000111700.12 | OATP1B3 | | | bile salts, organic anions |
| 175 | SLCO4C1 | ENSG00000173930.8 | | SLC21A20, OATP4C1, OATPX, OATP-H | | |
| 176 | SLCO1C1 | ENSG00000139155.8 | OATP1C1 | | | T4, T3, rT3 |
| 177 | SLC22A1 | ENSG00000175003.12 | 01-Oct | | F | organic cations |
| 178 | SLC22A2 | ENSG00000112499.12 | 02-Oct | | F | organic cations |
| 179 | SLC22A3 | ENSG00000146477.5 | 02-Oct | EMT (uptake-2 system) | F | organic cations |
| 180 | SLC22A4 | ENSG00000197208.5 | OCTN1 | ETT | C / Na+ | ergothioneine, zwitterions, organic cations |
| 181 | SLC22A5 | ENSG00000197375.12 | OCTN2 | CT1, CDSP | C / Na+, L-carnitine, F / organic cations | zwitterions (L-carnitine), organic cations |
| 182 | SLC22A6 | ENSG00000197901.11 | OAT1 | PAHT, NKT | E / organic anions | organic anions |
| 183 | SLC22A7 | ENSG00000137204.14 | OAT2 | NLT | F or E | organic anions |
| 184 | SLC22A8 | ENSG00000149452.15 | OAT3 | | E / dicarboxylate | organic anions |
| 185 | SLC22A9 | ENSG00000149742.9 | OAT7 | UST3, OAT4 | E / short chain fatty acids | organic anions |
| 186 | SLC22A10 | ENSG00000184999.11 | OAT5 | | O | not determined |
| 187 | SLC22A11 | ENSG00000168065.15 | OAT4 | | F or E | organic anions |
| 188 | SLC22A12 | ENSG00000197891.11 | URAT1 | RST, OAT4L | E / organic anions | urate, organic anions |
| 189 | SLC22A13 | ENSG00000172940.11 | OAT10 | ORCTL3, OCTL1 | E / anions | urate, organic anions |
| 190 | SLC22A14 | ENSG00000144671.10 | OCTL2 | ORCTL4 | O | not determined |
| 191 | SLC22A15 | ENSG00000163393.12 | FLIPT1 | | O | not determined |
| 192 | SLC22A16 | ENSG00000004809.13 | 39726 | FLIPT2, CT2 | F | L-carnitine, noncharged compounds |
| 193 | SLC22A17 | ENSG00000092096.14 | BOIT | BOCT, NGALR | O | not determined |
| 194 | SLC22A18 | ENSG00000110628.13 | | IMPT1, TSSC5, ORCTL2 | | probably organic anions |

| | | | | | | |
|---|---|---|---|---|---|---|
| 195 | SLC22A20 | ENSG00000197847.12 | OAT6 | | E / dicarboxylate | probably organic anions |
| 196 | SLC22A23 | ENSG00000137266.14 | | | O | |
| 197 | SLC22A24 | ENSG00000197658.9 | | | O | |
| 198 | SLC22A25 | ENSG00000196600.12 | UST6 | HIMTP | O | |
| 199 | SLC22A31 | ENSG00000259803.6 | | | | |
| 200 | SLC22A32 | ENSG00000109736.14 | MFSD10 | TETRAN | C / H+ | p-aminohippuric acid (PAH), indomethacin, diclofenac, mefenamic acid, etodolac [PMID: 18638446] |
| 201 | SLC22B1 | ENSG00000159164.9 | SV2A | | | Galactose [PMID: 25326386]; also binds levetiracetam [PMID: 15210974], selectracetam [PMID: 18183537], brivaracetam [PMID: 17785672]. |
| 202 | SLC22B2 | ENSG00000185518.11 | SV2B | | | |
| 203 | SLC22B3 | ENSG00000122012.13 | SV2C | | | |
| 204 | SLC22B4 | ENSG00000166111.9 | SVOP | | | Nicotinate [PMID: 21953179]; binds nucleotides in the TM region [PMID: 19390693] |
| 205 | SLC22B5 | ENSG00000157703.15 | SVOPL | | | |
| 206 | SLC23A1 | ENSG00000170482.16 | SVCT1 | YSPL3, NCBT2, MGC22361 | C / Na+ | L-ascorbic acid |
| 207 | SLC23A2 | ENSG00000089057.14 | SVCT2 | YSPL2, KIAA0238, NBTL1, NCBT1 | C / Na+ | L-ascorbic acid |
| 208 | SLC23A3 | ENSG00000213901.10 | SVCT3 | YSPL1, FLJ31168, E2BP3 | | |
| 209 | SLC24A1 | ENSG00000074621.13 | NCKX1 | RODX, HsT17412, KIAA0702 | E / 4 Na+, Ca2+, K+ | Na+, Ca2+, K+ |
| 210 | SLC24A2 | ENSG00000155886.11 | NCKX2 | | E / 4 Na+, Ca2+, K+ | Na+, Ca2+, K+ |
| 211 | SLC24A3 | ENSG00000185052.11 | NCKX3 | | E / 4 Na+, Ca2+, K+ | Na+, Ca2+, K+ |
| 212 | SLC24A4 | ENSG00000140090.17 | NCKX4 | SHEP6, FLJ38852 | E / 4 Na+, Ca2+, K+ | Na+, Ca2+, K+ |
| 213 | SLC24A5 | ENSG00000188467.10 | NCKX5 | JSX, SHEP4 | E / 4 Na+, Ca2+, K+ | Na+, Ca2+, K+ |
| 214 | SLC25A1 | ENSG00000100075.9 | CIC (citrate carrier) | CTP, tricarboxylate carrier, SLC20A3 | E / citrate-H+, malate | citrate, isocitrate, malate, PEP |
| 215 | SLC25A2 | ENSG00000120329.6 | ORC2 (ornithine carrier 2) | ORNT2 | E / ornithine, citrulline, H+; ornithine, H+ | ornithine, citrulline, lysine, arginine, histidine |
| 216 | SLC25A3 | ENSG00000075415.12 | PHC (phosphate carrier) | PTP, PiC | C / phosphate, H+; E / phosphate, OH- | phosphate |
| 217 | SLC25A4 | ENSG00000151729.10 | ANT1 (adenine | AAC1, T1, PEO2, | E / ADP, ATP | ADP, ATP |

| | | | | | | |
|---|---|---|---|---|---|---|
| | | | nucleotide translocase-1) | PEO3 | | |
| 218 | SLC25A5 | ENSG00000005022.5 | ANT2 (adenine nucleotide translocase-2) | AAC2, T2 | E / ADP, ATP | ADP, ATP |
| 219 | SLC25A5P1 | ENSG00000215347.3 | pseudogene | bK250D10, ANTP3 | | |
| 220 | SLC25A6 | ENSG00000169100.12 | ANT3 (adenine nucleotide translocase-3) | AAC3, T3 | E / ADP, ATP | ADP, ATP |
| 221 | SLC25A6P1 | ENSG00000233015.1 | pseudogene | | | |
| 222 | SLC25A7 | ENSG00000109424.3 | UCP1 (uncoupling protein 1) | thermogenin, proton carrier | F | H+ |
| 223 | SLC25A8 | ENSG00000175567.8 | UCP2 (uncoupling protein 2) | proton carrier, UCPH | F | H+ |
| 224 | SLC25A9 | ENSG00000175564.12 | UCP3 (uncoupling protein 3) | proton carrier | F | H+ |
| 225 | SLC25A10 | ENSG00000183048.11 | DIC (dicarboxylate carrier) | | E / malate, phosphate | malate, phosphate, succinate, sulphate, thiosulphate |
| 226 | SLC25A11 | ENSG00000108528.13 | OGC (oxoglutarate carrier) | oxoglutarate / malate carrier, SLC20A4 | E / oxoglutarate, malate | 2-oxoglutarate, malate |
| 227 | SLC25A12 | ENSG00000115840.13 | AGC1 (aspartate / glutamate carrier 1) | aralar1 | E / aspartate, glutamate, H+ | aspartate, glutamate |
| 228 | SLC25A13 | ENSG00000004864.13 | AGC2 (aspartate / glutamate carrier 2) | citrin, CTLN2, aralar2 | E / aspartate, glutamate, H+ | aspartate, glutamate |
| 229 | SLC25A14 | ENSG00000102078.15 | UCP5 (uncoupling protein 5) | BMCP1, brain mitochondrial carrier protein | O | |
| 230 | SLC25A15 | ENSG00000102743.14 | ORC1 (ornithine carrier 1) | ORNT1, HHH | E / ornithine, citrulline, H+; ornithine, H+ | ornithine, citrulline, lysine, arginine |
| 231 | SLC25A15P1 | ENSG00000227629.1 | pseudogene | | | |
| 232 | SLC25A16 | ENSG00000122912.14 | GDC (Graves' | GDA, ML7, HGT.1 | O | |

| | | | | | | |
|---|---|---|---|---|---|---|
| | | | disease carrier) | | | |
| 233 | SLC25A17 | ENSG00000100372.14 | | PMP34, ANC1, ANT1 | E / CoA; PAP, CoA, dPCoA; CoA,AMP; FAD, FMN; NAD+, AMP | CoA, FAD, NAD+, AMP, ADP, PAP, dPCoA, FMN |
| 234 | SLC25A18 | ENSG00000182902.13 | GC2 (glutamate carrier 2) | | C / glutamate, H+; E / glutamate, OH- | glutamate |
| 235 | SLC25A19 | ENSG00000125454.11 | DNC (deoxynucleotide carrier) | MUP1, TPC, MCPHA | E / thiamine pyrophosphate, thiamine monophosphate, thiamine; pyrophosphate, (deoxy)nucleotide | thiamine pyrophosphate, thiamine monophosphate, (deoxy)nucleotides |
| 236 | SLC25A20 | ENSG00000178537.9 | CAC (carnitine / acylcarnitine carrier) | CACT, carnitine carrier | E / carnitine, acylcarnitine; F (at slow rate) | carnitine, acylcarnitine |
| 237 | SLC25A20P1 | ENSG00000218363.1 | pseudogene | | | |
| 238 | SLC25A21 | ENSG00000183032.10 | ODC (oxoadipate carrier) | | E / oxoadipate, oxoglutarate | oxoadipate, oxoglutarate |
| 239 | SLC25A22 | ENSG00000177542.10 | GC1 (glutamate carrier 1) | | C / glutamate, H+; E / glutamate, OH- | glutamate |
| 240 | SLC25A23 | ENSG00000125648.14 | APC2 | ScaMC-3, MCSC2 | E / ATP-Mg, Pi | ATP-Mg2+, ATP, ADP, AMP, Pi |
| 241 | SLC25A24 | ENSG00000085491.15 | APC1 | ScaMC-1 | E / ATP-Mg, Pi | ATP-Mg2+, ATP, ADP, AMP, Pi |
| 242 | SLC25A25 | ENSG00000148339.12 | APC3 | ScaMC-2, MCSC, PCSCL | O | |
| 243 | SLC25A26 | ENSG00000144741.17 | SAMC | | E / S-adenosyl-methionine, S-adenosyl-homocysteine | S-adenosyl-methionine, S-adenosyl-homocysteine |
| 244 | SLC25A27 | ENSG00000153291.15 | UCP4 (uncoupling protein 4) | | O | |
| 245 | SLC25A28 | ENSG00000155287.10 | Mitoferrin 2 (Mfrn2) | MRS3 / 4, MRS4L | | Fe2+ |
| 246 | SLC25A29 | ENSG00000197119.12 | ORNT3 | CACL | | ornithine, acylcarnitine |
| 247 | SLC25A30 | ENSG00000174032.16 | | KMCP1, UCP6 | O | |
| 248 | SLC25A31 | ENSG00000151475.5 | AAC4, ANT4 (adenine nucleotide carrier 4) | SFEC | E / ADP, ATP | ADP, ATP |
| 249 | SLC25A32 | ENSG00000164933.11 | MFT | MFTC | | folate |

| | | | | | | |
|---|---|---|---|---|---|---|
| 250 | SLC25A33 | ENSG00000171612.6 | PNC1 (pyrimidine nucleotide carrier 1) | BMSC-MCP | | UTP |
| 251 | SLC25A34 | ENSG00000162461.7 | | | O | |
| 252 | SLC25A35 | ENSG00000125434.10 | | | O | |
| 253 | SLC25A36 | ENSG00000114120.11 | PNC2 (pyrimidine nucleotide carrier 2) | | | pyrimidine nucleotides |
| 254 | SLC25A37 | ENSG00000147454.13 | Mitoferrin 1 (Mfrn1) | HT015, MSC, MSCP | | $Fe^{2+}$ |
| 255 | SLC25A38 | ENSG00000144659.10 | | | | glycine ? |
| 256 | SLC25A39 | ENSG00000013306.15 | | CGI-69 | O | |
| 257 | SLC25A40 | ENSG00000075303.12 | | MCFP | O | |
| 258 | SLC25A41 | ENSG00000181240.13 | APC4 | SCaMC-3L | E | ATP-Mg / Pi |
| 259 | SLC25A42 | ENSG00000181035.13 | | | E | CoA, ADP, ATP, adenosine 3',5'-diphosphate, dPCoA |
| 260 | SLC25A43 | ENSG00000077713.18 | | | O | |
| 261 | SLC25A44 | ENSG00000160785.13 | | | O | |
| 262 | SLC25A45 | ENSG00000162241.12 | | | O | |
| 263 | SLC25A46 | ENSG00000164209.16 | | | O | |
| 264 | SLC25A47 | ENSG00000140107.10 | | C14orf68, HDMCP | O | |
| 265 | SLC25A48 | ENSG00000145832.12 | | FLJ44862 | O | |
| 266 | SLC25A49 | ENSG00000137409.18 | MTCH1 | MCH1 | O | |
| 267 | SLC25A50 | ENSG00000109919.9 | MTCH2 | MCH2 | O | |
| 268 | SLC25A51 | ENSG00000122696.12 | MCART1 | MCTR1 | O | |
| 269 | SLC25A51P1 | ENSG00000220483.4 | pseudogene | MCART3P | | |
| 270 | SLC25A51P2 | ENSG00000263716.1 | pseudogene | MCART4P | | |
| 271 | SLC25A51P3 | ENSG00000254181.1 | pseudogene | MCART5P | | |
| 272 | SLC25A52 | ENSG00000141437.8 | MCART2 | MCTR2 | O | |
| 273 | SLC25A53 | ENSG00000269743.2 | MCART6 | MCTR6 | O | |
| 274 | SLC26A1 | ENSG00000145217.13 | SAT1, SLC26A1 | | E / $SO_4^{2-}$, oxalate, | $SO_4^{2-}$, oxalate, glyoxylate |

| | | | | | | |
|---|---|---|---|---|---|---|
| | | | | | glyoxylate | |
| 275 | SLC26A2 | ENSG00000155850.7 | DTDST, SLC26A2 | | E / SO42-, oxalate, Cl- | SO42-, oxalate, Cl- |
| 276 | SLC26A3 | ENSG00000091138.12 | DRA, CLD, SLC26A3 | | E / Cl-, HCO3-, oxalate | Cl-, HCO3-, oxalate |
| 277 | SLC26A4 | ENSG00000091137.11 | PDS, Pendrin, SLC26A4 | | E / I-, Cl-, HCO3- | I-, Cl-, HCO3- |
| 278 | SLC26A5 | ENSG00000170615.14 | Prestin, SLC26A5 | | E / Cl-, formate, oxalate, SO42- | Cl-, formate, oxalate, SO42- |
| 279 | SLC26A6 | ENSG00000225697.10 | CFEX, PAT1, SLC26A6 | | E / Cl-, HCO3-, oxalate, OH-, formate | Cl-, HCO3-, oxalate, OH-, formate |
| 280 | SLC26A7 | ENSG00000147606.8 | SUT2, SLC26A7 | | E / Cl-, HCO3-, OH-, SO42-, Ch / Cl- | Cl-, HCO3-, OH-, SO42-, Ch: Cl- |
| 281 | SLC26A8 | ENSG00000112053.13 | TAT1, SLC26A8 | | E / Cl-, HCO3-, OH- | Cl-, HCO3-, OH- |
| 282 | SLC26A9 | ENSG00000174502.18 | SLC26A9 | | E / Cl-, HCO3, Ch / Cl-, HCO3- | Cl-, HCO3, Ch: Cl-, HCO3- |
| 283 | SLC26A10 | ENSG00000135502.16 | (SLC26A10) | | | |
| 284 | SLC26A11 | ENSG00000181045.14 | SUT1, KBAT, SLC26A11 | | E / Cl-, HCO3-, SO42-, oxalate, ?Ch / Cl- | Cl-, HCO3-, SO42-, oxalate (?), Ch: Cl- |
| 285 | SLC27A1 | ENSG00000130304.16 | FATP1 | FATP, ACSVL5 | LCFA transport, VLCFA activation | LCFA, VLCFA |
| 286 | SLC27A2 | ENSG00000140284.10 | FATP2 | ACSVL1, VLCS, VLACS, FACVL1 | LCFA transport, VLCFA activation | LCFA, VLCFA |
| 287 | SLC27A3 | ENSG00000143554.13 | FATP3 | ACSVL3, VLCS-3 | LCFA transport, VLCFA activation | LCFA, VLCFA |
| 288 | SLC27A4 | ENSG00000167114.12 | FATP4 | ACSVL4 | LCFA transport, VLCFA activation | LCFA, VLCFA |
| 289 | SLC27A5 | ENSG00000083807.9 | FATP5 | ACSVL6, VLCS-H2, VLACSR, FACVL3, BAL, ACSB, BACS | LCFA transport, bile acid conjugation | LCFA, bile acids |
| 290 | SLC27A6 | ENSG00000113396.12 | FATP6 | ACSVL2, VLCS-H1, FACVL2 | LCFA transport, VLCFA activation | LCFA, VLCFA |
| 291 | SLC28A1 | ENSG00000156222.11 | CNT1 | | concentrative 1:1 sodium:nucleoside | |
| 292 | SLC28A2 | ENSG00000137860.11 | CNT2 | | concentrative 1:1 sodium:nucleoside | |

| | | | | | | |
|---|---|---|---|---|---|---|
| 293 | SLC28A3 | ENSG00000197506.7 | CNT3 | | concentrative 2:1 sodium: nucleoside 1:1 proton: nucleoside probably 1:1:1 sodium:proton:nucleoside | |
| 294 | SLC29A1 | ENSG00000112759.16 | ENT1 | | F | |
| 295 | SLC29A2 | ENSG00000174669.11 | ENT2 | | F | |
| 296 | SLC29A3 | ENSG00000198246.7 | ENT3 | | unclear, possibly proton-linked | |
| 297 | SLC29A4 | ENSG00000164638.10 | ENT4 | | unclear, possibly proton-linked | |
| 298 | SLC30A1 | ENSG00000170385.9 | | ZnT1 | | |
| 299 | SLC30A2 | ENSG00000158014.14 | | ZnT2 | | |
| 300 | SLC30A3 | ENSG00000115194.10 | | ZnT3 | | |
| 301 | SLC30A4 | ENSG00000104154.6 | | ZnT4 | | |
| 302 | SLC30A5 | ENSG00000145740.18 | | ZnT5 | | |
| 303 | SLC30A6 | ENSG00000152683.14 | | ZnT6 | | |
| 304 | SLC30A7 | ENSG00000162695.11 | | ZnT7 | | |
| 305 | SLC30A8 | ENSG00000164756.12 | | ZnT8 | | |
| 306 | SLC30A9 | ENSG00000014824.13 | | ZnT9 | | |
| 307 | SLC30A10 | ENSG00000196660.10 | | ZnT10 | | |
| 308 | SLC31A1 | ENSG00000136868.10 | CTR1 | hCtr1, Ctr1, COPT1 | energy in-dependent, potassium-dependent | copper (I), cisplatin |
| 309 | SLC31A1P1 | ENSG00000224426.1 | pseudogene | SLC31A1P, CTR1psi, CTR1P | | |
| 310 | SLC31A2 | ENSG00000136867.10 | CTR2 | hCtr2, Ctr2, COPT2 | unknown | copper, cisplatin |
| 311 | SLC32A1 | ENSG00000101438.3 | VIAAT | VGAT | E / H+ | GABA / glycine |
| 312 | SLC33A1 | ENSG00000169359.13 | ACATN1 | AT-1 | F | acetyl-CoA |
| 313 | SLC33A2 | ENSG00000167700.8 | MFSD3 | | | |
| 314 | SLC34A1 | ENSG00000131183.10 | NaPi-IIa | Napi-3, NPT2, npt2 | C / Na, HPO42- | inorganic phosphate (divalent) |
| 315 | SLC34A2 | ENSG00000157765.11 | NaPi-IIb | | C / Na, HPO42- | inorganic phosphate (divalent) |
| 316 | SLC34A3 | ENSG00000198569.9 | NaPi-IIc | | C / Na, HPO42- | inorganic phosphate (divalent) |
| 317 | SLC35A1 | ENSG00000164414.16 | CST | CMPST | E / CMP | CMP-sialic acid |
| 318 | SLC35A2 | ENSG00000102100.14 | UGT | UGAT, UGTL, UGALT, UGT1, UGT2 | E / UMP | UDP-galactose, UDP-N-acetylgalactosamine |

| | | | | | | |
|---|---|---|---|---|---|---|
| 319 | SLC35A3 | ENSG00000117620.12 | | DKFZp781P1297 | E / UMP | UDP-N-acetylglucosamine |
| 320 | SLC35A4 | ENSG00000176087.14 | | MGC2541 | | |
| 321 | SLC35A5 | ENSG00000138459.8 | | FLJ11130, FLJ20730, FLJ25973, DKFZp434E102 | | |
| 322 | SLC35B1 | ENSG00000121073.13 | | UGTREL1 | | |
| 323 | SLC35B2 | ENSG00000157593.16 | PAPST1 | UGTrel4, SLL | | PAPS |
| 324 | SLC35B3 | ENSG00000124786.9 | PAPST2 | CGI-19, C6orf196 | | PAPS |
| 325 | SLC35B4 | ENSG00000205060.10 | YEA | YEA4, FLJ14697 | | UDP-xylose, UDP-N-acetylglucosamine |
| 326 | SLC35C1 | ENSG00000181830.8 | FUCT1 | FLJ11320, FLJ14841 | E / GMP | GDP-fucose |
| 327 | SLC35C2 | ENSG00000080189.14 | OVCOV1 | CGI-15, C20orf5, FLJ37039, FLJ46434, MGC20633, MGC32079, MGC39183, BA394O2.1 | | GDP-fucose (?) |
| 328 | SLC35D1 | ENSG00000116704.7 | UGTREL7 | KIAA0260, MGC138236 | E / UMP | UDP-glucuronic acid, UDP-N-acetylgalactosamine |
| 329 | SLC35D2 | ENSG00000130958.11 | HFRC1 | UGTrel8, Hfrc, SQV7L, MGC117215, MGC142139 | | UDP-N-acetylglucosamine |
| 330 | SLC35D3 | ENSG00000182747.4 | FRCL1 | MGC102873, bA55K22.3 | | |
| 331 | SLC35D4 | ENSG00000134490.13 | TMEM241 | C18orf45 | | |
| 332 | SLC35E1 | ENSG00000127526.13 | | FLJ14251, FLJ36689, MGC44954, DKFZp564G0462 | | |
| 333 | SLC35E2 | ENSG00000215790.6 | | FLJ34996, FLJ44537, KIAA0447, MGC104754, MGC117254, MGC126715, MGC138494, DKFZp686M0869 | | |
| 334 | SLC35E3 | ENSG00000175782.9 | | BLOV1 | | |
| 335 | SLC35E4 | ENSG00000100036.12 | | MGC129826 | | |
| 336 | SLC35F1 | ENSG00000196376.10 | | C6orf169, FLJ13018, dJ230I3.1 | | |
| 337 | SLC35F2 | ENSG00000110660.14 | | HSNOV1, FLJ13018, DKFZp667H1615 | | |
| 338 | SLC35F3 | ENSG00000183780.12 | | FLJ37712 | | |
| 339 | SLC35F4 | ENSG00000151812.14 | | C14orf36, FLJ37712, c14_5373 | | |
| 340 | SLC35F5 | ENSG00000115084.12 | | FLJ22004 | | |
| 341 | SLC35F6 | ENSG00000213699.8 | C2orf18 | ANT2BP | | |
| 342 | SLC35G1 | ENSG00000176273.14 | | TMEM20, C10orf60 | | |
| 343 | SLC35G2 | ENSG00000168917.8 | | TMEM22 | | |
| 344 | SLC35G3 | ENSG00000164729.7 | | AMAC1, TMEM21A | | |

| | | | | | | |
|---|---|---|---|---|---|---|
| 345 | SLC35G4 | ENSG00000236396.6 | | AMAC1L1 | | |
| 346 | SLC35G5 | ENSG00000177710.5 | | AMAC, AMAC1L2 | | |
| 347 | SLC35G6 | ENSG00000259224.2 | | AMAC1L3, TMEM21B | | |
| 348 | SLC36A1 | ENSG00000123643.12 | PAT1 | LYAAT1 | C / H+ | GABA, P, G, beta-alanine |
| 349 | SLC36A2 | ENSG00000186335.8 | PAT2 | tamodorin 1 | C / H+ | P, G, A, hydroxyproline |
| 350 | SLC36A3 | ENSG00000186334.9 | PAT3 | | | |
| 351 | SLC36A4 | ENSG00000180773.14 | PAT4 | LYAAT2 | C / H+ | P, tryptophan |
| 352 | SLC37A1 | ENSG00000160190.13 | SLC37A1, SPX1 | glucose-6-phosphate | E / inorganic phosphate | |
| 353 | SLC37A2 | ENSG00000134955.11 | SLC37A2, SPX2 | glucose-6-phosphate | E / inorganic phosphate | |
| 354 | SLC37A3 | ENSG00000157800.17 | SLC37A3, SPX3 | unknown | | |
| 355 | SLC37A4 | ENSG00000137700.16 | SLC37A4, G6PT, SPX4 | glucose-6-phosphate | E / inorganic phosphate | |
| 356 | SLC38A1 | ENSG00000111371.15 | SNAT1 | ATA1, NAT2, SAT1 | C / Na+ | Q, A, N, C, H, S |
| 357 | SLC38A2 | ENSG00000134294.13 | SNAT2 | ATA2, SAT2 | C / Na+ | A, N, C, Q, G, H, M, P, S |
| 358 | SLC38A3 | ENSG00000188338.14 | SNAT3 | SN1 | C / Na+, E / H+ | Q, H, A, N |
| 359 | SLC38A4 | ENSG00000139209.15 | SNAT4 | ATA3, NAT3, PAAT | C / Na+ | A, N, C, G, S, T |
| 360 | SLC38A5 | ENSG00000017483.14 | SNAT5 | SN2 | C / Na+, E / H+ | Q, N, H, S |
| 361 | SLC38A6 | ENSG00000139974.15 | SNAT6 | | | |
| 362 | SLC38A7 | ENSG00000103042.8 | SNAT7 | | ? / Na+ | Q, H, S ,A, N |
| 363 | SLC38A8 | ENSG00000166558.10 | SNAT8 | | ? / Na+ | Q, H, A, N [PMID: 25451601] |
| 364 | SLC38A9 | ENSG00000177058.11 | N/A | | | R, Q, H, P, K, E, L |
| 365 | SLC38A10 | ENSG00000157637.12 | N/A | | | |
| 366 | SLC38A11 | ENSG00000169507.9 | N/A | | | |
| 367 | SLC39A1 | ENSG00000143570.17 | ZIP1, ZIRTL | | | Zn |
| 368 | SLC39A2 | ENSG00000165794.9 | ZIP2, Eti-1, 6A1 | | | Zn |
| 369 | SLC39A3 | ENSG00000141873.10 | ZIP3 | | | Zn, not specific |
| 370 | SLC39A4 | ENSG00000147804.9 | ZIP4 | | | Zn |
| 371 | SLC39A5 | ENSG00000139540.11 | ZIP5, LZT-Hs7 | | | Zn |

| | | | | | | |
|---|---|---|---|---|---|---|
| 372 | SLC39A6 | ENSG00000141424.12 | ZIP6, LIV-1 | | | Zn |
| 373 | SLC39A7 | ENSG00000112473.16 | ZIP7, HKE4, RING5 | | | Zn, Mn |
| 374 | SLC39A8 | ENSG00000138821.12 | ZIP8, BIGM103, LZT-Hs6 | | | Zn, Cd, Mn |
| 375 | SLC39A9 | ENSG00000029364.11 | ZIP9 | | | |
| 376 | SLC39A10 | ENSG00000196950.13 | ZIP10, LZT-Hs2 | | | Zn |
| 377 | SLC39A11 | ENSG00000133195.11 | ZIP11 | | | |
| 378 | SLC39A12 | ENSG00000148482.11 | ZIP12j, LZT-Hs8 | | | Zn |
| 379 | SLC39A13 | ENSG00000165915.13 | ZIP13, LZT-Hs9 | | | Zn |
| 380 | SLC39A14 | ENSG00000104635.13 | ZIP14, LZT-Hs4 | | | Zn, Fe, Mn, Cd |
| 381 | SLC40A1 | ENSG00000138449.10 | ferroportin (FPN1) | MTP1, IREG1 | F ? | ferrous iron |
| 382 | SLC41A1 | ENSG00000133065.10 | MgtE | | Ch | Mg2+ (Sr2+, Zn2+, Cu2+, Fe2+, Co2+, Ba2+, Cd2+) |
| 383 | SLC41A2 | ENSG00000136052.9 | | SLC41A1-L1, SLC41A1-like 1 | Ch | Mg2+ (Ba2+, Ni2+, Co2+, Fe2+, Mn2+) |
| 384 | SLC41A3 | ENSG00000114544.15 | | SLC41A1-L2, SLC41A1-like 2, FLJ20473 | Ch? (predicted based on homology with the other 2 members/MgtE transporter) | |
| 385 | SLC42A1 | ENSG00000112077.15 | RhAG | RHAG (Rh50A) | H+ | NH4+, NH3 |
| 386 | SLC42A2 | ENSG00000132677.12 | RhBG | RHBG | electrogenic, no coupled ions | NH4+, NH3, methyl amine, methyl ammonium |
| 387 | SLC42A3 | ENSG00000140519.12 | RhCG | RHCG (RhGK) | electroneutral, possibly H+ | NH4+, NH3 |
| 388 | SLC43A1 | ENSG00000149150.8 | LAT3 | POV1 | F | L-BCAAs, amino alcohols |
| 389 | SLC43A2 | ENSG00000167703.14 | LAT4 | | F | L-BCAAs, amino alcohols |
| 390 | SLC43A3 | ENSG00000134802.17 | EEG1 | FOAP-13 | O | |
| 391 | SLC44A1 | ENSG00000070214.15 | CTL1 | CDw92, CHTL1 | E? | choline |
| 392 | SLC44A2 | ENSG00000129353.14 | CTL2 | PP1292 | O | choline |
| 393 | SLC44A3 | ENSG00000143036.16 | CTL3 | MGC45474 | O | |
| 394 | SLC44A4 | ENSG00000204385.10 | CTL4 | NG22, FLJ14491 | O | |
| 395 | SLC44A5 | ENSG00000137968.16 | CTL5 | MGC34032 | O | |
| 396 | SLC45A1 | ENSG00000162426.14 | SLC45A1 | Past-A, DNB5 | C / H+ | glucose, galactose, sucrose |
| 397 | SLC45A2 | ENSG00000164175.14 | SLC45A2 | MATP, AIM1, | C / H+ [PMID: 25164149] | sucrose, glucose, fructose [PMID: 25164149] |

| | | | | | | |
|---|---|---|---|---|---|---|
| | | | | underwhite | | |
| 398 | SLC45A3 | ENSG00000158715.5 | SLC45A3 | Prostein | C / H+ [PMID: 25164149] | sucrose, glucose, fructose [PMID: 25164149] |
| 399 | SLC45A4 | ENSG00000022567.9 | SLC45A4 | KIAA1126 | C / H+ [PMID: 25164149] | sucrose, glucose, fructose [PMID: 25164149] |
| 400 | SLC46A1 | ENSG00000076351.12 | PCFT | HCP1 | C / proton-symporter | Reduced folates, folic acid, antifolates |
| 401 | SLC46A2 | ENSG00000119457.7 | TSCOT | Ly110 | | unknown |
| 402 | SLC46A3 | ENSG00000139508.14 | | | | Uncertain; lysosomal export of maytansine conjugates |
| 403 | SLC47A1 | ENSG00000142494.13 | | MATE1 | | tetraethylammonium (TEA), 1-methyl-4-phenylpyridinium (MPP), cimetidine, metformin, guanidine, procainamide, cephalexin, cephradine |
| 404 | SLC47A2 | ENSG00000180638.17 | | MATE2-K (MATE2, MATE2-B) | | TEA, MPP, cimetidine, metformin, guanidine, procainamide |
| 405 | SLC48A1 | ENSG00000211584.13 | HRG-1 | | C / H+ ? | heme |
| 406 | SLC49A1 | ENSG00000162769.12 | FLVCR1 | FLVCR, MFSD7B, AXPC1, PCARP | unknown | heme |
| 407 | SLC49A2 | ENSG00000119686.9 | FLVCR2 | MFSD7C, CCT, EPV, PVHH, FLVCRL14q | unknown | heme |
| 408 | SLC49A3 | ENSG00000169026.12 | MFSD7 | LP2561, FLJ22269 | unknown | unknown |
| 409 | SLC49A4 | ENSG00000138463.8 | DIRC2 | RCC4, FLJ14784 | unknown | unknown |
| 410 | SLC50A1 | ENSG00000169241.17 | RAG1AP | HsSWEET1 | F | glucose |
| 411 | SLC51A | ENSG00000163959.9 | OST alpha | OST alpha-OST beta | F | bile acids |
| 412 | SLC51B | ENSG00000186198.3 | OST beta | | | steroids |
| 413 | SLC52A1 | ENSG00000132517.14 | | RFVT1, RFT1, GPR172B, PERV-A receptor 2, GPCR42, PAR2, FLJ10060 | Na+, Cl-, pH-independent | |
| 414 | SLC52A2 | ENSG00000185803.8 | | RFVT2, RFT3, GPR172A, PERV-A receptor 1, GPCR41, PAR1, D15Ertd747e, FLJ11856 | Na+, Cl-, pH-independent | |

| | | | | | | |
|---|---|---|---|---|---|---|
| 415 | SLC52A3 | ENSG00000101276.14 | | RFVT3, RFT2, C20orf54, RIKEN cDNA 2310046K01, hypothetical protein LOC113278, bA371L19.1 | C / Na+, Cl-indep., pH sensitive | |
| 416 | SLC53A1 | ENSG00000143324.13 | XPR1 | SYG1, XR | | Phosphate [PMID: 23791524] |
| 417 | SLC54A1 | ENSG00000060762.18 | MPC1 | BRP44L | | Pyruvate [PMID: 22628554] [PMID: 22628558] |
| 418 | SLC54A2 | ENSG00000143158.10 | MPC2 | BRP44 | | Pyruvate [PMID: 22628558] |
| 419 | SLC54A3 | ENSG00000238205.2 | MPC1L | | | Pyruvate [PMID: 27317664] |
| 420 | SLC55A1 | ENSG00000168924.14 | LETM1 | Mdm38 homolog (yeast) | E / Ca2+:H+[PMID: 19797662] [PMID: 27669901], E / K+:H+ [PMID: 15138253] [PMID: 17925330] | Ca2+, K+, H+ (K+: [PMID: 20197279] [PMID: 15138253], [PMID: 17925330], yeast protein: [PMID: 17541427]), arguments against Ca2+ transport [PMID: 20197279]. |
| 421 | SLC55A2 | ENSG00000165046.12 | LETM2 | FLJ25409 | | |
| 422 | SLC55A3 | ENSG00000050426.15 | LETMD1 | HCCR, HCCR-1, HCCR-2 | | |
| 423 | SLC56A1 | ENSG00000164466.12 | SFXN1 | FLJ12876 | | Probably falsely identified as a tricarboxylate carrier in [PMID: 8132491] as discussed in [PMID: 11274051]. Likely transports pyridoxin or another heme precursor or ALAS2 cofactor [PMID: 11274051] [PMID: 12670026]. |
| 424 | SLC56A2 | ENSG00000156398.12 | SFXN2 | | | |
| 425 | SLC56A3 | ENSG00000107819.13 | SFXN3 | | | |
| 426 | SLC56A4 | ENSG00000183605.16 | SFXN4 | | | |
| 427 | SLC56A5 | ENSG00000144040.12 | SFXN5 | | | |
| 428 | SLC57A1 | ENSG00000170113.15 | NIPA1 | | | Mg2+ [PMID: 17166836], also Sr2+, Fe2+ and Co2+ to a lesser extent [PMID: 18667602] |
| 429 | SLC57A2 | ENSG00000140157.14 | NIPA2 | | | Mg2+ [PMID: 18667602] |
| 430 | SLC57A3 | ENSG00000163293.11 | NIPAL1 | NIPA3 [PMID: 18667602] | | Mg2+, Sr2+, Ba2+, Fe2+, Cu2+ [PMID: 18667602] |
| 431 | SLC57A4 | ENSG00000104361.9 | NIPAL2 | NIPA4 [PMID: 18667602] | | Mg2+, Sr2+, Ba2+[PMID: 18667602] |
| 432 | SLC57A5 | ENSG00000001461.16 | NIPAL3 | NPAL3 | | |
| 433 | SLC57A6 | ENSG00000172548.14 | NIPAL4 | ichthyin, NIPA4 | | |

| | | | | | | |
|---|---|---|---|---|---|---|
| 434 | SLC58A1 | ENSG00000102158.19 | MAGT1 | | Channel-like [PMID: 19940067] | Mg2+ [PMID: 15804357] |
| 435 | SLC58A2 | ENSG00000104723.20 | TUSC3 | N33 | | Mg2+, Fe2+, Cu2+, Mn2+ [PMID: 18667602] [PMID: 19940067] |
| 436 | SLC59A1 | ENSG00000168389.17 | MFSD2A | MFSD2 | C / LPC:Na+, uptake [PMID: 24828044] | LPC (lysophosphatidylcholine) form of DHA (docosahexaenoic acid) [PMID: 24828044] |
| 437 | SLC59A2 | ENSG00000205639.9 | MFSD2B | | | |
| 438 | SLC60A1 | ENSG00000174514.12 | MFSD4A | | | |
| 439 | SLC61A1 | ENSG00000182544.8 | MFSD5 | | | Molybdate [PMID: 21464289] |
| 440 | SLC62A1 | ENSG00000154122.12 | ANKH | | | Pyrophosphate [PMID: 10894769] |
| 441 | SLC63A1 | ENSG00000169682.17 | SPNS1 | | | |
| 442 | SLC63A2 | ENSG00000183018.8 | SPNS2 | | | Phosphorylated sphingolipids [PMID: 19074308]; phosphorylated Fingolimod (FTY720-P) [PMID: 21084291]; sphingosine-1-phosphate (S1P), dihydrosphingosine-1-phosphate (DH-S1P), phyto-S1P, C17-S1P [PMID: 21084291] |
| 443 | SLC63A3 | ENSG00000182557.7 | SPNS3 | | | |
| 444 | SLC64A1 | ENSG00000134851.12 | TMEM165 | | E / Ca2+:H+ | |
| 445 | SLC65A1 | ENSG00000141458.12 | NPC1 | | | Cholesterol [PMID: 17989073] [PMID: 17989072] [PMID: 27410046] |
| 446 | SLC65A2 | ENSG00000015520.14 | NPC1L1 | | | Cholesterol [PMID: 14976318] |

* Abbreviations for transport type: C: Cotransporter; E: Exchanger; F: Facilitated transporter; O: Orphan transporter.
